# Associations between commuting modes and risk of 16 site-specific cancers in the UK Biobank

**DOI:** 10.1093/ije/dyaf117

**Published:** 2025-07-08

**Authors:** Win Thu, Alana Cavadino, Alistair Woodward, Sandar Tin Tin

**Affiliations:** Epidemiology and Biostatistics, School of Population Health, University of Auckland, Auckland, New Zealand; Epidemiology and Biostatistics, School of Population Health, University of Auckland, Auckland, New Zealand; Epidemiology and Biostatistics, School of Population Health, University of Auckland, Auckland, New Zealand; Epidemiology and Biostatistics, School of Population Health, University of Auckland, Auckland, New Zealand; Cancer Epidemiology Unit, Oxford Population Health, University of Oxford

**Keywords:** active modes, commuting, site-specific cancers, prospective cohort study

## Abstract

**Background:**

The choice of transport mode may influence cancer risk by affecting physical activity level, sedentary behaviour, and exposure to environmental pollution. This study investigated the associations between commuting modes and 16 site-specific cancers in the UK Biobank.

**Methods:**

The UK Biobank is a prospective cohort study involving about 500 000 participants. Information on transport modes was collected at recruitment, and incident cancer cases were identified through linkage to national cancer registries. Multivariable Cox proportional hazards models were used.

**Results:**

There were 252 334 employed participants included, and 15 828 incident cancer cases were identified over a median follow-up of 11.7 years. Compared to the car-only mode, cycling (exclusively or combined with any other modes) was associated with a lower risk of colon [hazard ratio (HR): 0.72; 95% confidence interval: 0.53–0.96], renal (HR: 0.60; 0.38–0.96), and stomach (HR: 0.27; 0.10–0.71) cancers. Walking (exclusively or combined with any motorized mode) was associated with a lower risk of renal (HR: 0.67; 0.49–0.92) and liver (HR: 0.55; 0.31–0.98) cancers. Public transport users were less engaged in other physical activities, and its use was associated with a higher risk of bladder cancer (HR: 1.39; 1.01–1.90).

**Conclusions:**

Active commuting, even combined with motorized modes, is associated with a lower risk of some common cancers.

Key messages

- There is limited evidence on how active commuting modes, in comparison to car use, are associated with the risks of site-specific cancers.
- In this analysis of the UK Biobank data, active modes, even combined with motorized modes, are associated with a lower risk of colon, renal, liver, and stomach cancers.
- Promoting and supporting active commuting can be an important intervention for cancer prevention.

## Background

Cancer is a leading cause of mortality globally, with the World Health Organization estimating nearly 10 million deaths (approximately one in six deaths) in 2020 [1]. Physical activity has been associated with a lower risk of over 20 cancers [2–4], whereas sedentary behaviour, independent of physical activity, is linked to a higher cancer risk [5, 6].

Active commuting, such as walking and cycling, integrates physical activity into daily routines, while using private vehicles increases sedentary behaviour and environmental pollution [7]. Public transport may promote physical activity, as users need to walk or cycle to stops or transit points [8]. Our recent meta-analysis, involving 27 studies, showed an inverse association between transport-related physical activity and the risk of colorectal, breast, and endometrial cancers, but evidence for other cancer sites is currently limited [9]. The associations between commuting mode and cancer risk may also be influenced by broader environmental factors such as air pollution and green spaces [10–15].

This study investigated the associations between commuting modes and 16 site-specific cancers in the UK Biobank, taking environmental factors into account.

## Methods

### Study design and participants

The UK Biobank is a prospective cohort study involving around 500 000 participants aged between 40 and 69 years recruited across the UK between 2006 and 2010. At baseline, sociodemographic, lifestyle, employment, medical, and health-related information were collected, and participants were followed up through linkage to national health records [16]. We included employed participants who reported their commuting mode/s but excluded those who had prevalent malignant cancers or benign central nervous system tumours, or missing/unknown commute mode, or who withdrew from the study. We also excluded women who reported hysterectomy (removal of the uterus) and bilateral oophorectomy (removal of both ovaries) from analyses for endometrial and ovarian cancers, respectively.

### Exposure (commuting modes)

Participants in paid employment or self-employed, except those who indicated they always work from home, were asked, “What types of transport do you use to get to and from work?”. They could select one or more of the five options: car/motor vehicle, walk, public transport, cycle, and mode other than those. Since many participants used more than one mode and the number of participants who used an active mode (walking or cycling) alone was small, we grouped the modes into four overall categories: car/motor vehicle only, walk (walk only or combined with other modes except cycling), cycle (cycle only or combined with any other modes), and public transport (public transport only or combined with car).

### Outcomes (site-specific cancers)

We included 16 incident invasive cancers (oesophageal adenocarcinoma, gastric, colon, rectal, hepatic, pancreas, renal, bladder, thyroid, multiple myeloma, bronchus and lung, and malignant melanoma in both genders; breast, endometrial, and ovarian cancers in women; and prostate in men). The first 15 cancers have been associated with either physical activity, body weight, or exposure to environmental factors [2, 3]. We included prostate cancer as some studies indicated cycling could be associated with a higher level of prostate-specific antigen, a commonly used biomarker to detect cancer [17, 18] although its association with physical activity is inconclusive [19, 20]. The endpoints were the first incident cancer diagnosis or cancer first recorded in death certificates and identified from cancer and death registry data using ICD-10 codes (Supplementary Table S1). The linked data were available up to 31 December 2020, 30 November 2021, and 31 December 2016 for participants in England, Scotland, and Wales, respectively.

### Covariates

These include sociodemographic factors: age at baseline, gender, assessment centre, Townsend deprivation index, ethnicity, education, household income; lifestyle factors: smoking, alcohol drinking, vegetable and processed meat intake; body mass index; other physical activities: stair climbing, walking for pleasure, strenuous sports, other exercises, light and heavy DIY (do-it-yourself), walking or standing at job, physical or heavy manual work at job; TV and not work-related computer time; modes used for non-commute trips (same categorizations as commute trips); and environmental factors: residential particulate matter, particles with a diameter less than 2.5 microns (millionths of a metre) (PM_2.5_) and nitrogen dioxide (NO_2_) levels, green space percentage (Table 1, Supplementary Table S2). Residential PM_2.5_ and NO_2_ levels [measured in 2010 as microgram per cubic metre (µg/m^3^)] were estimated from a land use regression model [21, 22] and greenspace as a proportion of all land use types in a circular distance buffer of 1000 m was derived from the Generalized Land Use Database for England.

**Table 1.** Baseline characteristics of the sample by commuting mode

**Characteristics** ^a^	**Car only** (*n* = 158 805)	**Walk** ^b^ (*n* = 36 282)	**Cycle** ^c^ (*n* = 19 329)	**Public transport** ^d^ (*n* = 37 918)
	*n* (%)	*n* (%)	*n* (%)	*n* (%)
Age (mean, SD)	53 (6.9)	53 (6.9)	51 (6.8)	53 (7.1)
Gender				
Women	79 442 (50.0)	23 541 (64.9)	6774 (35.0)	21 648 (57.1)
Household income (£) per year				
<18 000	12 758 (8.0)	4906 (13.5)	1356 (7.0)	4654 (12.3)
18 000–30 999	31 558 (19.9)	7812 (21.5)	3245 (16.8)	7771 (20.5)
31 000–51 999	47 806 (30.1)	9454 (26.1)	5656 (29.3)	9637 (25.4)
≥52 000	52 017 (32.8)	10 374 (28.6)	7885 (40.8)	12 020 (31.7)
Unknown	14 666 (9.2)	3736 (10.3)	1187 (6.1)	3836 (10.1)
Smoking				
Non-smoker	66 107 (41.6)	15 612 (43.0)	7485 (38.7)	15 983 (42.2)
Previous smoker	74 903 (47.2)	16 912 (46.6)	10 098 (52.2)	17 124 (45.2)
Current smoker (<15 cigarettes/day)	4968 (3.1)	1180 (3.3)	521 (2.7)	1536 (4.1)
Current smoker (>15 cigarettes/day)	6832 (4.3)	1270 (3.5)	318 (1.6)	1762 (4.6)
Current smoker (unknown quantity)	5561 (3.5)	1198 (3.3)	871 (4.5)	1393 (3.7)
Unknown	434 (0.3)	110 (0.3)	36 (0.2)	120 (0.3)
Alcohol (intake frequency)				
Daily/almost daily	30 127 (19.0)	6481 (17.9)	4282 (22.2)	6998 (18.5)
3–4 times a week	39 331 (24.8)	8480 (23.4)	5645 (29.2)	8628 (22.8)
1–2 times a week	45 687 (28.8)	9785 (27.0)	5154 (26.7)	10 006 (26.4)
1–3 times a month	19 039 (12.0)	4637 (12.8)	2010 (10.4)	4626 (12.2)
Special occasions only	15 469 (9.7)	4200 (11.6)	1374 (7.1)	4668 (12.3)
Never	9059 (5.7)	2674 (7.4)	853 (4.4)	2947 (7.8)
Unknown	93 (0.1)	25 (0.1)	11 (0.1)	45 (0.1)
Vegetable (cooked and raw, tablespoons/day)				
<3	31 921 (20.1)	6469 (17.8)	3002 (15.5)	7941 (20.9)
3–4	58 380 (36.8)	12 917 (35.6)	7275 (37.6)	13 635 (36.0)
5–6	38 663 (24.3)	9342 (25.7)	4913 (25.4)	8828 (23.3)
>6	28 919 (18.2)	7344 (20.2)	4047 (20.9)	7219 (19.0)
Unknown	922 (0.6)	210 (0.6)	92 (0.5)	295 (0.8)
Processed meat (frequency per week)				
Never	13 113 (8.3)	4262 (11.7)	2562 (13.3)	4176 (11.0)
<1	46 827 (29.5)	11 620 (32.0)	5180 (26.8)	11 525 (30.4)
1	46 856 (29.5)	10 033 (27.7)	5464 (28.3)	10 421 (27.5)
>1	51 771 (32.6)	10 307 (28.4)	6102 (31.6)	11 677 (30.8)
Unknown	238 (0.1)	60 (0.2)	21 (0.1)	119 (0.3)
Other exercise (min/day)				
0	80 243 (50.5)	19 284 (53.2)	3891 (20.1)	21 065 (55.6)
1–30	17 001 (10.7)	3721 (10.3)	3697 (19.1)	4037 (10.6)
31–60	34 509 (21.7)	7748 (21.4)	6748 (34.9)	7573 (20.0)
61–90	17 051 (10.7)	3697 (10.2)	2923 (15.1)	3473 (9.2)
91–>180	9489 (6.0)	1692 (4.7)	1975 (10.2)	1641 (4.3)
Unknown	512 (0.3)	140 (0.4)	95 (0.5)	129 (0.3)
TV and computer time (hour/day)				
<3	54 433 (34.3)	14 268 (39.3)	9463 (49.0)	13 728 (36.2)
3–4	63 666 (40.1)	13 757 (37.9)	6571 (34.0)	14 536 (38.3)
>4	40 522 (25.5)	8216 (22.6)	3278 (17.0)	9595 (25.3)
Unknown	184 (0.1)	41 (0.1)	17 (0.1)	59 (0.2)
Distance between home and work (mile)				
<1	6090 (3.8)	16 005 (44.1)	2448 (12.7)	953 (2.5)
2–5	51 525 (32.4)	12 156 (33.5)	10 396 (53.8)	13 038 (34.4)
6–10	41 705 (26.3)	2740 (7.6)	3921 (20.3)	10 096 (26.6)
>10	51 480 (32.4)	3400 (9.4)	2076 (10.7)	9433 (24.9)
Unknown/not provided mode	8005 (5.0)	1981 (5.5)	488 (2.5)	4398 (11.6)
Transport mode^e^ (non-commute trips)				
Car only	92 062 (58.0)	7435 (20.5)	3493 (18.1)	8013 (21.1)
Walk	54 345 (34.2)	24 440 (67.4)	4360 (22.6)	17 905 (47.2)
Cycle	6759 (4.3)	1315 (3.6)	10 790 (55.8)	1432 (3.8)
Public transport	5139 (3.2)	3002 (8.3)	669 (3.5)	10 493 (27.7)
Unknown/not provided mode	500 (0.3)	90 (0.2)	17 (0.1)	75 (0.2)
BMI				
<18.5	577 (0.4)	242 (0.7)	122 (0.6)	214 (0.6)
18.5–24.9	48 040 (30.3)	13 802 (38.0)	8480 (43.9)	12 434 (32.8)
25–25.9	67 326 (42.4)	14 157 (39.0)	7935 (41.1)	15 238 (40.2)
≥30	39 723 (25.0)	7399 (20.4)	2428 (12.6)	9239 (24.4)
Unknown	3139 (2.0)	682 (1.9)	364 (1.9)	793 (2.1)

BMI, body mass index.

a All values are *n* (%) except age.

b Walking only or combined it with either car or public transport or both.

c Cycling only or combined it with any other mode/s.

d Public transport alone or combined it with car.

e Similar to the commute modes categorizations.

### Statistical analyses

We used multivariable Cox proportional hazards models with a diagnosis of the cancer of interest as the failure variable, diagnosis of any other cancer, death or end of follow-up as censored observations, and attained age as the underlying time scale. Hazard ratios (HRs) and 95% confidence intervals (95% CIs) compared other commuting modes to car-only mode. We stratified models by age group, gender, assessment centre, and Townsend deprivation index and adjusted for the covariates mentioned above. For female-specific cancers (breast, endometrial, and ovarian), we additionally adjusted for menopausal status, parity, and use of hormone replacement therapy and oral contraceptive pills (time in years since the last used). We handled missing data (<2% for most categorical variables) by using the missing indicator method. We used the Schoenfeld formal residual test to check the proportionality assumption.

We conducted subgroup analyses by gender (male, female) and smoking status (never and ever) for cancers with more than 10 cases in each exposure group. We assessed effect modification by including interaction terms in the models and used a likelihood ratio test to compare the models with and without the interaction terms. For breast, colorectal, and bronchus and lung cancers, which were previously associated with environmental factors [10–15], we also conducted subgroup analyses by PM_2.5_, NO_2_, and greenspace, using the World Health Organization’s recommended thresholds of 15 and 25 µg/m^3^ for PM_2.5_ and NO_2_, and the median of the sample for greenspace. To check for potential reverse causation, we conducted a sensitivity analysis by removing the first four years of follow-up for all results. Further, we conducted sensitivity analyses by additionally adjusting for the presence or absence of long-standing illness, and for specific modes (i.e. car only, walking only, cycling only, and public transport only) with common cancers. We used R statistical software (version 4.2.3) for all analyses [23].

## Results

**Figure 1.**
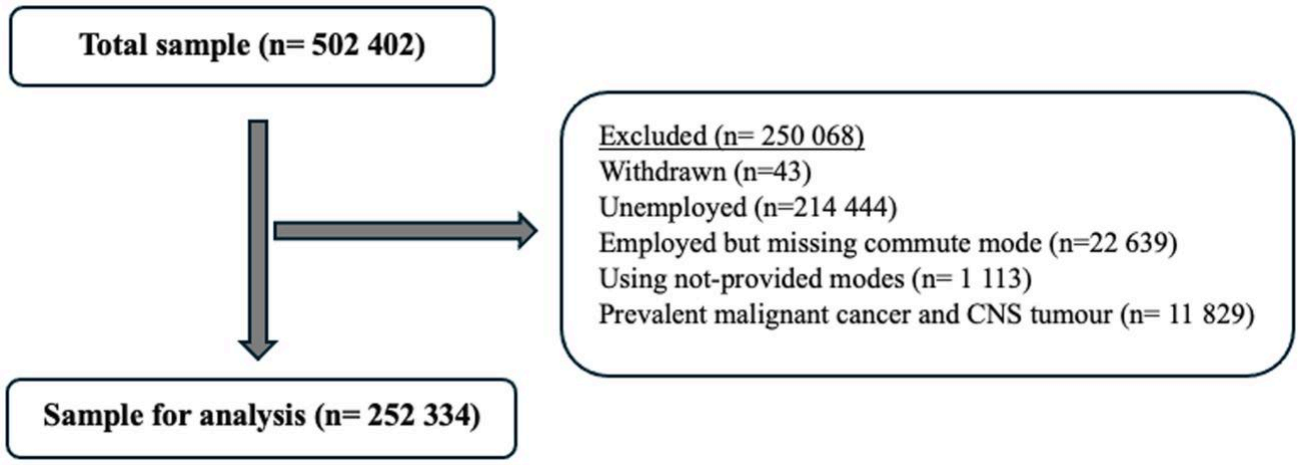
Study sample selection.

Of the 252 334 participants included in the analyses (Fig. 1), around two-thirds (63%) used car only, followed by public transport (15%), walking (14%), and cycling (8%). Participant characteristics by commuting mode are presented in Table 1 and Supplementary Table S3.

### Cancers (both genders)

**Figure 2.**
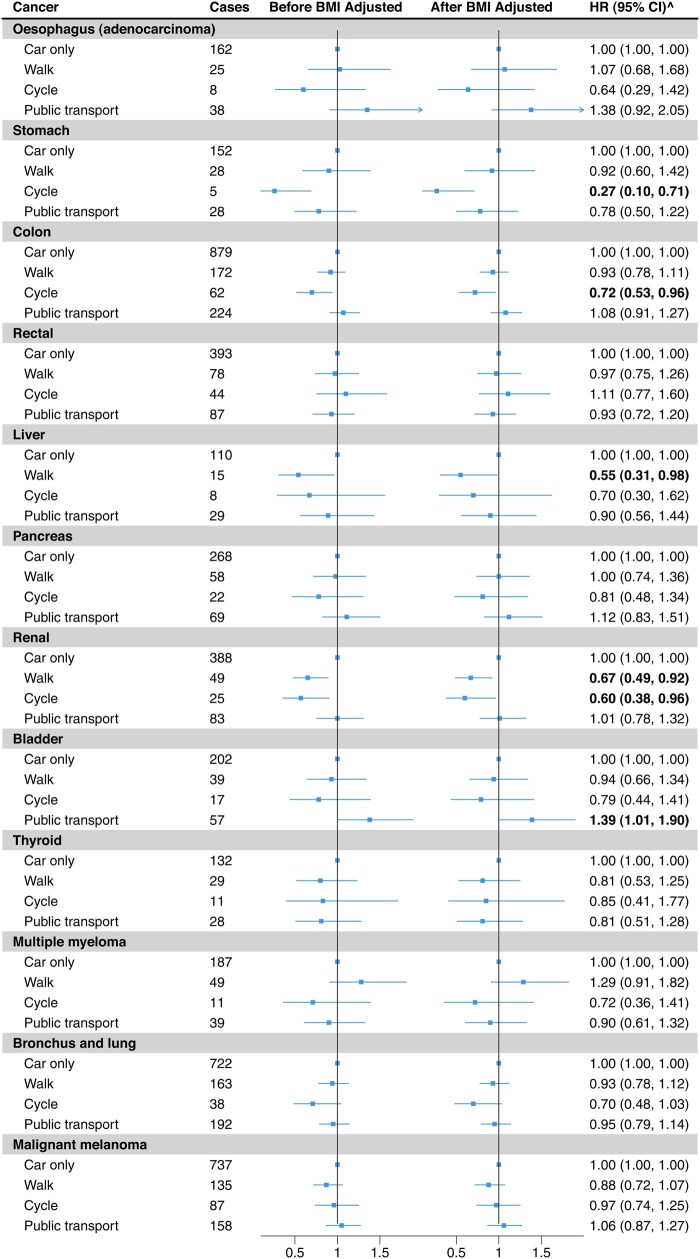
Estimates of the associations between commuting modes and risks of site-specific cancers. Horizontal lines show site-specific cancers, the number of cancer cases for each commuting mode, effect estimates (HRs) and 95% confidence intervals for the associations of commuting modes (walking, cycling, and public transport in comparison to car-only) with each cancer. Left vertical line shows the associations before BMI adjustment, and the right vertical line shows the associations after BMI adjustment. Stratified by age group, gender, assessment centre, and Townsend deprivation index. Adjusted for education, smoking status, alcohol intake frequency, vegetable intake, meat intake, stairs climbed frequency, walking for pleasure, DIY light, DIY heavy, other exercises, strenuous sports, walking/standing and manual/physical at job, TV and non-work computer time, non-commute mode, and environmental factors. BMI, body mass index; ^after BMI adjustment.

During the median follow-up of 11.7 years, there were 6542 incident cancers (Table 2). In the fully adjusted model, compared to car-only mode, walking was associated with a lower risk of renal (HR: 0.67; 95% CI: 0.49–0.92) and liver cancers (HR: 0.55; 0.31–0.98) and cycling with a lower risk of stomach (HR: 0.27; 0.10–0.71), colon (HR: 0.72; 0.53–0.96), and renal (HR: 0.60; 0.38–0.96) cancers. Public transport was associated with a higher risk of bladder cancer (HR: 1.39; 1.01–1.90) (Fig. 2, Supplementary Table S4).

**Table 2.** Number of cancers (total, women, men)

Cancer	Number of cancers
Both genders	
Oesophagus (adenocarcinoma)	
Total	233
Women	33
Men	200
Stomach	
Total	213
Women	63
Men	150
Colon	
Total	1337
Women	601
Men	736
Rectal	
Total	602
Women	214
Men	388
Liver	
Total	162
Women	60
Men	102
Pancreas	
Total	417
Women	186
Men	231
Renal	
Total	545
Women	186
Men	359
Bladder	
Total	315
Women	67
Men	248
Thyroid	
Total	200
Women	152
Men	48
Multiple myeloma	
Total	286
Women	127
Men	159
Bronchus and lung	
Total	1115
Women	555
Men	560
Malignant melanoma	
Total	1117
Women	547
Men	570
Gender-specific cancer	
Women only	
Breast	4200
Endometrial	582
Ovarian	369
Men only	
Prostate	4135

### Gender-specific cancers

**Figure 3.**
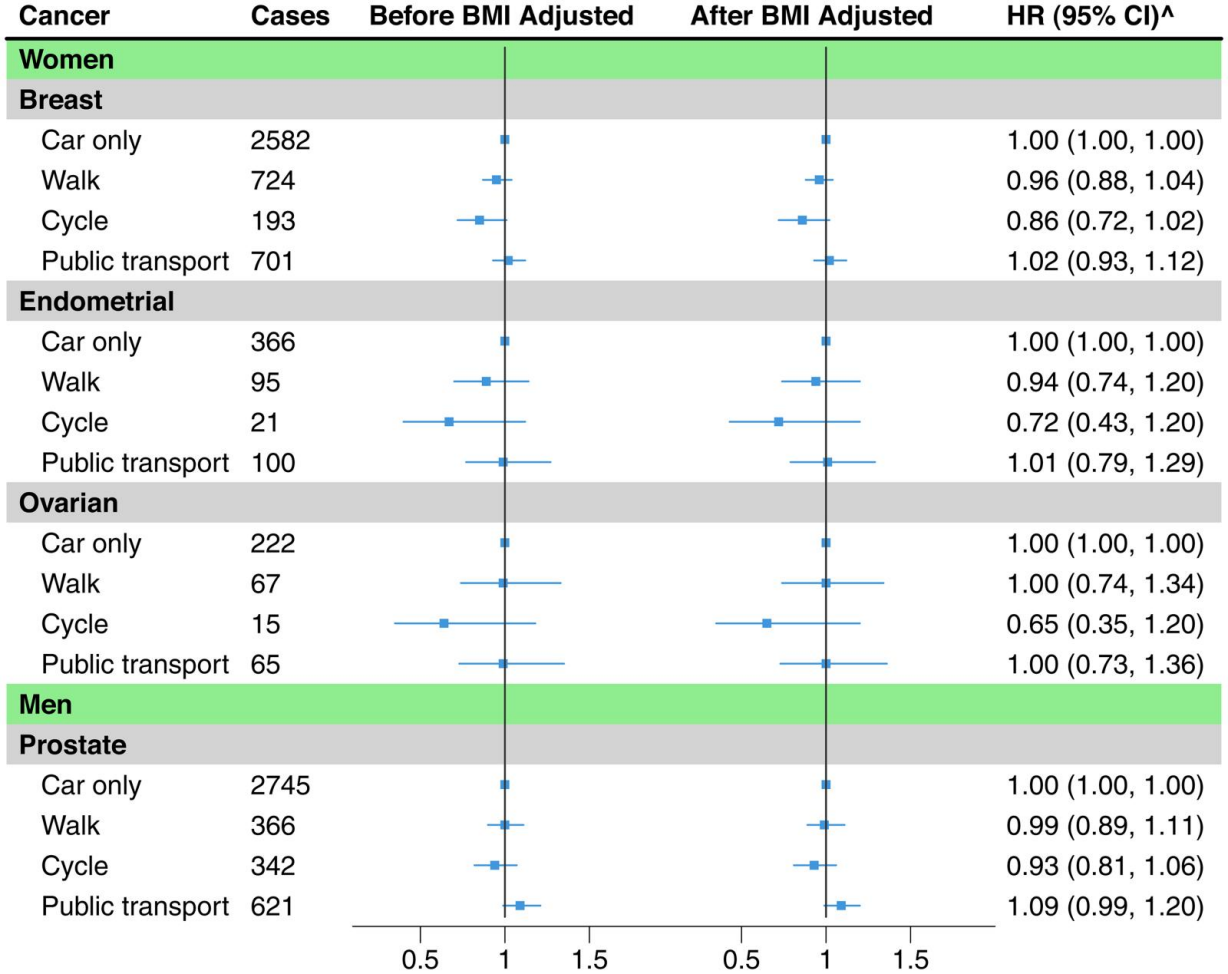
Estimates of the associations between commuting modes and risks of gender-specific cancers. Horizontal lines show site-specific cancers, the number of cancer cases for each commuting mode, effect estimates (HRs) and 95% confidence intervals for the associations of commuting modes (walking, cycling, and public transport in comparison to car-only) with each cancer. Left vertical line shows the associations before BMI adjustment, and the right vertical line shows the associations after BMI adjustment. Stratified by age group, assessment centre, and Townsend deprivation index. Adjusted education, smoking status, alcohol intake frequency, vegetable intake, meat intake, stairs climbed frequency, walking for pleasure, DIY light, DIY heavy, other exercises, strenuous sports, walking/standing and manual/physical at job, TV and non-work computer time, non-commute mode, and environmental factors. For female-specific cancers, menopausal status, parity, hormonal therapy, and oral contraceptives use were additionally adjusted. BMI, body mass index; ^after BMI adjustment.

Of the 131 405 women included, 5151 developed breast, endometrial, or ovarian cancer (Table 2). There was no significant association between commuting modes and risks of female-specific cancers (Fig. 3 and Supplementary Table S5). Additional adjustment for menopausal status, parity, and uses of hormone replacement therapy and oral contraceptive pills did not affect the results (Supplementary Table S6). Of the 120 929 men included, 4135 participants developed prostate cancer (Table 2). There was no association between commuting modes and prostate cancer risk (Fig. 3 and Supplementary Table S7).

### Subgroup analyses by gender, smoking, and environmental factors

There was no effect modification by gender for non-gender-specific cancers (Supplementary Table S8) or smoking status (Supplementary Table S9). Results were similar in subgroup analyses by environmental factors (PM_2.5_, NO_2_, and greenspace) for colon, bronchus and lung, and breast cancers (Supplementary Tables S10–S12).

### Individual-specific modes

A total of 13 561 participants walked exclusively, 5698 cycled exclusively, and 22 706 used public transport exclusively. No significant association with individual-specific modes was observed for colon, bronchus and lung, malignant melanoma, breast (women) and prostate (men) cancers (Supplementary Table S13).

### Sensitivity analyses

Similar associations were observed after removing the first four years of follow-up (Supplementary Table S14), and after adjusting for the presence or absence of a long-standing illness (Supplementary Table S15).

## Discussion

In this analysis involving 252 334 participants and 15 828 incident cancer cases, compared to using a car exclusively, cycling was associated with a lower risk of colon, renal and stomach cancers, and walking with a lower risk of renal and liver cancers.

Our findings add to the existing evidence in this area. Previous research has consistently reported an inverse association between active commuting, particularly cycling, and overall cancer incidence and mortality [24, 25]. A relatively smaller number of studies have assessed the risk of site-specific cancers with different levels of each active mode. In our previous meta-analysis, involving 22 studies published up to February 2023, there was an inverse association between active modes and the risk of colorectal, breast, and endometrial cancers (5%, 1%, and 9% lower risk respectively per 10 metabolic equivalent of task hour increment in transport-related physical activity per week). The associations for cycling are likely to be stronger as the intensity is greater, but the evidence on the risk of site-specific cancers associated with each active mode (walking and cycling separately), compared to a private motorised mode, is currently limited.

In this analysis, we compared each active mode with the car-only mode and observed a significant association of cycling, but not walking, with a lower risk of colon cancer. While we did not observe a significant association of cycling or walking with female-specific cancers, there tended to be a lower risk associated with cycling, particularly for breast cancer. A previous analysis of the UK Biobank data, however, did not observe any significant association of active patterns of commuting with colon or breast cancer incidence or mortality [26] possibly due to a shorter duration of follow-up (with smaller numbers of cases) or possibly because the risk associated with cycling and walking was not assessed separately. A previous analysis of the UK Biobank data found that public transport use, compared to regular automobile use, was associated with a higher risk of lung cancer in areas with a higher residential NO_2_ level [27], which we did not observe, possibly due to differences in exposure categorization and the NO_2_ threshold that was applied. The previous study categorized exposure groups based on the frequency of trips, but our study did not. The previous study used the study median of the NO_2_ level as a threshold for subgroup analyses, whereas our study used the WHO-recommended threshold for NO_2_.

In addition to colon and breast cancers, we found a lower risk of renal and stomach cancers associated with cycling and a lower risk of renal and liver cancers with walking. These cancers have also been associated with physical activity in general or leisure time physical activity [28–30]. While we did not observe a significant association with other physical activity-related cancers such as oesophageal adenocarcinoma, pancreatic cancer, bladder cancer, and multiple myeloma, there tended to be a lower risk particularly with cycling, which may be confirmed in future studies with a larger sample of cyclists.

Active commuting, by increasing physical activity level, may be associated with a lower cancer risk through various mechanisms, including improvement in immune function, reduction in systemic inflammation, modulation of circulating levels of sex hormones, reduction of body weight and effects on mechanical processes such as increasing colon motility and reducing the contact time between mucosa cells and carcinogens [31–35]. Despite evidence suggesting that active mode users might be exposed to higher doses of air pollution [36], exercise may outweigh the adverse effects of air pollution [37]; we found a lower risk of colon cancer with cycling in the areas with residential air pollution levels higher than the recommended thresholds.

To our knowledge, this is the first analysis that investigated the associations of commuting modes with 16 site-specific cancers. The sample size is relatively large, and we were able to control for important confounders at the individual level as well as at the broader environmental level. Our analysis, however, has limitations. First, we were only able to assess the cancer risk associated with mixed commuting modes (due to the limited number of participants and cases for exclusive walking and cycling modes); however, we ensured that each exposure group had a predominant mode (e.g. for the walking group, we combined walking only with car or public transport, not with cycling) to facilitate a better interpretation of the results. We also conducted mode-specific sensitivity analyses to assess the associations with each specific mode. Second, the number of cases for some cancers was small, particularly in the cycling group, which required cautious interpretations of the findings. Third, self-reported commuting mode collected at recruitment may not be accurate. Self-selection to commuting mode may also affect the findings, for example, sun-sensitive participants may not be as likely to walk or cycle, which may have resulted in the null findings for melanoma. We were also not able to account for potential changes in the commuting status or modes over time, for instance, some participants may retire or change workplace during follow-up. Fourth, the age of cancer onset could vary by site. This variation, together with the older age of study participants, the wide age range, and the long progression time of many cancers, could affect the interpretation of the findings. For example, participants may have already developed preclinical cancers. To address this, we conducted sensitivity analyses by excluding the first four years of follow-up and observed similar findings. Because many cancers progress over decades, it is also important to consider commuting modes used earlier in life, which this study could not capture as the relevant data were not available. Fifth, the environmental factors measured at residential levels may not accurately reflect the exposure levels along the commuting routes. Lastly, the study used data from the UK, a high-income country, with predominantly white participants, which may limit the generalizability of the findings to other contexts.

## Conclusion

Active commuting, even if combined with motorized modes, was associated with a lower risk of some common cancers, with stronger associations observed for cycling. Promoting and supporting the use of active modes for commuting could be an effective lifestyle intervention to prevent cancer and provide other health and environmental benefits.

## Supplementary Material

dyaf117_Supplementary_Data

## Data Availability

The data used in the study can be accessed through the application and approval from the UK Biobank.
