# Supplementary material for: Associations between commuting modes and risk of 16 site-specific cancers in the UK Biobank
Source: Int J Epidemiol. 2025 Jul 8;54(4):dyaf117. doi: 10.1093/ije/dyaf117 (PMC12237514; doi:10.1093/ije/dyaf117)
Supplement: dyaf117_Supplementary_Data [file dyaf117_supplementary_data.docx]

**Supplementary material**

**List of Tables**

[Table S1: ICD10 codes for cancers included 2](#_Toc201085352)

[Table S2: Covariates and data fields in the UK Biobank dataset 2](#_Toc201085353)

[Table S3: Baseline characteristics of the sample by commuting mode 3](#_Toc201085354)

[Table S4: Results for cancers in both genders 5](#_Toc201085355)

[Table S5: Results for female-specific cancers 7](#_Toc201085356)

[Table S6: Results for female-specific cancers by additionally adjusting for reproductive health factors 7](#_Toc201085357)

[Table S7: Results for male-specific cancer (prostate cancer) 7](#_Toc201085358)

[Table S8: Results for subgroup analyses by gender for cancers with >10 cases per exposure group 8](#_Toc201085359)

[Table S9: Results for subgroup analyses by smoking status for cancers with >10 cases per exposure group 8](#_Toc201085360)

[Table S10: Results for subgroup analyses by PM_2.5_ levels 9](#_Toc201085361)

[Table S11: Results for subgroup analyses by NO_2_ levels 9](#_Toc201085362)

[Table S12: Results for subgroup analyses by residential greenspace levels 10](#_Toc201085363)

[Table S13: Results for cancers in both genders (individual specific modes) 10](#_Toc201085364)

[Table S14: Results for analyses by removing the first four years of follow-up 11](#_Toc201085365)

[Table S15: Sensitivity analyses by adjusting long-standing illness additionally 11](#_Toc201085366)

Table S1: ICD10 codes for cancers included

| **Cancer** | **ICD 10 codes** |
| --- | --- |
| Breast | "C504" "C509" "C501" "C503" "C505" "C508" "C502" "C500" "C506" |
| Prostate | C61 |
| Bronchus and lung | "C341" "C343" "C340" "C349" "C342" "C348" |
| Malignant Melanoma | "C433" "C437" "C436" "C435" "C434" "C439" "C432" "C431" "C430" |
| Endometrial | "C541" "C542" "C549" "C543" "C540" |
| Renal | C64 |
| Colon | "C184" "C180" "C183" "C187" "C186" "C182" "C185" "C189" "C181" "C188" |
| Rectal | "C200" "C201" "C202" "C203" "C204" "C205" "C206" "C207" "C208" "C209" |
| Ovarian | C54 |
| Multiple myeloma | C900 |
| Bladder | "C679" "C672" "C671" "C675" "C676" "C674" "C673" "C678" "C670" "C677" |
| Oesophagus | "C153" "C155" "C154" "C159" "C151" "C158" "C150" "C152" |
| Pancreas | "C251" "C250" "C259" "C253" "C254" "C252" "C258" "C257" |
| Liver | "C220" "C23" "C221" "C229" "C223" "C227" "C224" |
| Stomach | "C163" "C169" "C160" "C165" "C161" "C162" "C168" "C164" "C166" |
| Thyroid | C73 |

Table S2: Covariates and data fields in the UK Biobank dataset

| **Covariates** | **Data field name** | **Data field number** |
| --- | --- | --- |
| Gender | Sex | 31 |
| Ethnicity | Ethnic | 21000 |
| Education | Qualifications | 6138 |
| Household income | Average household income before tax | 738 |
| Smoking | Current tobacco smoking, Past tobacco smoking, number of cigarettes smoke | 1239;1249; 3456 |
| Alcohol | Alcohol intake frequency | 1558 |
| Vegetable | Salad/raw vegetable intake + Cooked vegetable intake | 1299;1289 |
| Processed meat | Processed meat intake | 1349 |
| Walk for pleasure | Last 4 week PA, Duration of walking for pleasure | 6164, 981 |
| Strenous sports | Last 4 week PA, Duration of strenous sports | 6164, 1001 |
| Other exercise | Last 4 week PA, Duration of other exercises | 6164, 3647 |
| DIY Light | Last 4 week PA, Duration of light DIY | 6164, 1021 |
| DIY heavy | Last 4 week PA, Duration of heavy DIY | 6164, 2634 |
| Stairs climbed | Frequency of stair climbing in last 4 weeks | 943 |
| Walking/standing at job | Job involves mainly walking or standing | 806 |
| Heavy manual/physical at job | Job involves heavy manual or physical work | 816 |
| TV time | Time spent watching television (TV) | 1070 |
| Computer time | Time spent using computer | 1080 |
| Distance between home and work | Distance between home and job workplace | 796 |
| Non-commute mode | Types of transport used (excluding work) | 6162 |
| Trip frequency | Frequency of travelling from home to job workplace | 777 |
| PM2.5 level | Particulate matter air pollution (pm2.5); 2010 | 24006 |
| NO2 level | Nitrogen dioxide air pollution; 2010 | 24003 |
| Greenspace | Greenspace percentage, buffer 1000m | 24500 |
| Long standing illness | Long-standing illness, disability or infirmity | 2188 |
| Menopausal status | Had menopause | 2724 |
| Parity | Number of live births | 2734 |
| Use of HRT | Female specific factors | 2814, 3546 |
| Use of OC pills | Female specific factors | 2784, 2804 |
| BMI |  |  |

Table S3: Baseline characteristics of the sample by commuting mode

| **Characteristics^a^** | **Car only** (n=158 805) | **Walk**^b^ (n=36 282) | **Cycle**^c^ (n=19 329) | **Public Transport**^d^ (n=37 918) |
| --- | --- | --- | --- | --- |
|  | n (%) | n (%) | n (%) | n (%) |
| Ethnicity |  |  |  |  |
| White | 150391 (94.7) | 33847 (93.3) | 18592 (96.2) | 33243 (87.7) |
| Asian | 3860 (2.4) | 904 (2.5) | 182 (0.9) | 1400 (3.7) |
| Caribbean, African | 2157 (1.4) | 719 (2.0) | 173 (0.9) | 1920 (5.1) |
| Mixed | 921 (0.6) | 299 (0.8) | 162 (0.8) | 413 (1.1) |
| Unknown | 1476 (0.9) | 513 (1.4) | 220 (1.1) | 942 (2.5) |
| Education |  |  |  |  |
| University/College | 53365 (33.6) | 14343 (39.5) | 10737 (55.5) | 15598 (41.1) |
| GCE A/O level | 55153 (34.7) | 12448 (34.3) | 5006 (25.9) | 12515 (33.0) |
| Other professional qualifications | 32820 (20.7) | 5515 (15.2) | 2481 (12.8) | 5761 (15.2) |
| Unknown | 17467 (11.0) | 3976 (11.0) | 1105 (5.7) | 4044 (10.7) |
| Stairs climbed (frequency per day) |  |  |  |  |
| None | 9493 (6.0) | 2386 (6.6) | 926 (4.8) | 2738 (7.2) |
| 1-5 times a day | 34726 (21.9) | 7869 (21.7) | 3625 (18.8) | 9429 (24.9) |
| 6-10 times a day | 63323 (39.9) | 13514 (37.2) | 7560 (39.1) | 13849 (36.5) |
| 11-15 times a day | 28654 (18.0) | 6614 (18.2) | 4055 (21.0) | 6212 (16.4) |
| More than 16 times a day | 21966 (13.8) | 5723 (15.8) | 3107 (16.1) | 5459 (14.4) |
| Unknown | 643 (0.4) | 176 (0.5) | 56 (0.3) | 231 (0.6) |
| Walk for pleasure (min/day) |  |  |  |  |
| 0 | 49414 (31.1) | 9510 (26.2) | 5206 (26.9) | 12394 (32.7) |
| 1-30 | 28525 (18.0) | 5586 (15.4) | 2624 (13.6) | 6713 (17.7) |
| 31-60 | 40825 (25.7) | 9921 (27.3) | 5141 (26.6) | 9443 (24.9) |
| 61-90 | 18299 (11.5) | 5081 (14.0) | 2685 (13.9) | 4286 (11.3) |
| 91 - >180 | 21342 (13.4) | 6053 (16.7) | 3623 (18.7) | 4934 (13.0) |
| Unknown | 400 (0.3) | 131 (0.4) | 50 (0.3) | 148 (0.4) |
| Strenuous sports (min/day) |  |  |  |  |
| 0 | 138134 (87.0) | 32394 (89.3) | 14017 (72.5) | 33974 (89.6) |
| 1-30 | 1694 (1.1) | 343 (0.9) | 350 (1.8) | 308 (0.8) |
| 31-60 | 8808 (5.5) | 1732 (4.8) | 2303 (11.9) | 1753 (4.6) |
| 61-90 | 6268 (3.9) | 1093 (3.0) | 1528 (7.9) | 1122 (3.0) |
| 91 - >180 | 3606 (2.3) | 638 (1.8) | 1088 (5.6) | 668 (1.8) |
| Unknown | 295 (0.2) | 82 (0.2) | 43 (0.2) | 93 (0.2) |
| DIY light (min/day) |  |  |  |  |
| 0 | 79554 (50.1) | 17893 (49.3) | 9018 (46.7) | 20736 (54.7) |
| 1-30 | 21865 (13.8) | 5539 (15.3) | 2867 (14.8) | 5222 (13.8) |
| 31-60 | 24773 (15.6) | 6022 (16.6) | 3389 (17.5) | 5448 (14.4) |
| 61-90 | 13198 (8.3) | 2803 (7.7) | 1736 (9.0) | 2670 (7.0) |
| 91 - >180 | 17482 (11.0) | 3371 (9.3) | 2133 (11.0) | 3362 (8.9) |
| Unknown | 1933 (1.2) | 654 (1.8) | 186 (1.0) | 480 (1.3) |
| DIY heavy (min/day) |  |  |  |  |
| 0 | 95363 (60.1) | 23908 (65.9) | 11094 (57.4) | 26099 (68.8) |
| 1-30 | 13639 (8.6) | 2814 (7.8) | 1761 (9.1) | 2619 (6.9) |
| 31-60 | 16371 (10.3) | 3304 (9.1) | 2203 (11.4) | 3000 (7.9) |
| 61-90 | 10544 (6.6) | 2011 (5.5) | 1395 (7.2) | 1987 (5.2) |
| 91 - >180 | 19431 (12.2) | 3277 (9.0) | 2511 (13.0) | 3450 (9.1) |
| Unknown | 3457 (2.2) | 968 (2.7) | 365 (1.9) | 763 (2.0) |
| Walking/Standing at Job (frequency) |  |  |  |  |
| Never/rarely | 50860 (32.0) | 12740 (35.1) | 7186 (37.2) | 15989 (42.2) |
| Sometimes | 49711 (31.3) | 10351 (28.5) | 5964 (30.9) | 11107 (29.3) |
| Usually | 25587 (16.1) | 5095 (14.0) | 2925 (15.1) | 4148 (10.9) |
| Always | 32514 (20.5) | 8082 (22.3) | 3242 (16.8) | 6621 (17.5) |
| Unknown | 133 (0.1) | 14 (0.0) | 12 (0.1) | 53 (0.1) |
| Heavy Manual/Physical at Job (frequency) | |  |  |  |
| Never/rarely | 98595 (62.1) | 23999 (66.1) | 12681 (65.6) | 27504 (72.5) |
| Sometimes | 35757 (22.5) | 7838 (21.6) | 4139 (21.4) | 6682 (17.6) |
| Usually | 12171 (7.7) | 2191 (6.0) | 1348 (7.0) | 1724 (4.5) |
| Always | 12177 (7.7) | 2215 (6.1) | 1156 (6.0) | 1955 (5.2) |
| Unknown | 105 (0.1) | 39 (0.1) | 5 (0.0) | 53 (0.1) |
| Trip frequency (outward trip) per week | |  |  |  |
| 2 and less | 11908 (7.5) | 2755 (7.6) | 1058 (5.5) | 3956 (10.4) |
| 3-4 | 36434 (22.9) | 9058 (25.0) | 4328 (22.4) | 9262 (24.4) |
| 5 | 94465 (59.5) | 21208 (58.5) | 12217 (63.2) | 22107 (58.3) |
| >5 | 14390 (9.1) | 2810 (7.7) | 1574 (8.1) | 2172 (5.7) |
| Unknown/Not provided mode | 1608 (1.0) | 451 (1.2) | 152 (0.8) | 421 (1.1) |
| PM_2.5_  level |  |  |  |  |
| Below the WHO’s  recommended threshold | 85152 (53.6) | 13094 (36.1) | 8022 (41.5) | 13732 (36.2) |
| Above the threshold | 62040 (39.1) | 19522 (53.8) | 9637 (49.9) | 19614 (51.7) |
| Unknown | 11613 (7.3) | 3666 (10.1) | 1670 (8.6) | 4572 (12.1) |
| NO_2_ level | |  |  |  |
| Below the WHO’s  recommended threshold | 77087 (48.5) | 10330 (28.5) | 6555 (33.9) | 10336 (27.3) |
| Above the threshold | 79997 (50.4) | 25163 (69.4) | 12448 (64.4) | 26721 (70.5) |
| Unknown | 1721 (1.1) | 789 (2.2) | 326 (1.7) | 861 (2.3) |
| Greenspace % | |  |  |  |
| Below the sample’s median | 58232 (36.7) | 20883 (57.6) | 10567 (54.7) | 21350 (56.3) |
| Above the median | 80623 (50.8) | 10560 (29.1) | 6450 (33.4) | 11107 (29.3) |
| Unknown | 19950 (12.6) | 4839 (13.3) | 2312 (12.0) | 5461 (14.4) |

^a^all values are n (%) except age

^b^walking only or combined it with either car or public transport or both, ^c^cycling only or combined it with any other mode/s

^d^public transport alone or combined it with car

Table S4: Results for cancers in both genders

|  | **Hazard ratio (95% CI)** | | | |
| --- | --- | --- | --- | --- |
|  | **Car only (ref)** | **Walk**^a^ | **Cycle**^b^ | **Public Transport**^c^ |
| **Oesophagus (adenocarcinoma)** |  |  |  |  |
| Model 0 | 1.00 | 0.92 (0.60, 1.41) | 0.43 (0.21, 0.87) | 1.18 (0.82, 1.70) |
| Model 1 | 1.00 | 0.94 (0.61, 1.45) | 0.45 (0.22, 0.93) | 1.23 (0.85, 1.78) |
| Model 2 | 1.00 | 0.96 (0.62, 1.48) | 0.53 (0.26, 1.11) | 1.24 (0.86, 1.80) |
| Model 3 | 1.00 | 1.05 (0.67, 1.64) | 0.61 (0.28, 1.35) | 1.35 (0.91, 2.01) |
| Model 4 | 1.00 | 1.03 (0.66, 1.62) | 0.60 (0.27, 1.33) | 1.35 (0.91, 2.01) |
| Model 5 | 1.00 | 1.07 (0.68, 1.68) | 0.64 (0.29, 1.42) | 1.38 (0.92, 2.05) |
| **Stomach** |  |  |  |  |
| Model 0 | 1.00 | 0.87 (0.58, 1.32) | 0.26 (0.11, 0.64) | 0.75 (0.50, 1.14) |
| Model 1 | 1.00 | 0.89 (0.59, 1.35) | 0.27 (0.11, 0.67) | 0.77 (0.51, 1.17) |
| Model 2 | 1.00 | 0.93 (0.61, 1.41) | 0.30 (0.12, 0.74) | 0.80 (0.53, 1.23) |
| Model 3 | 1.00 | 0.91 (0.59, 1.40) | 0.27 (0.10, 0.69) | 0.79 (0.50, 1.23) |
| Model 4 | 1.00 | 0.90 (0.59, 1.39) | 0.26 (0.10, 0.69) | 0.78 (0.50, 1.22) |
| Model 5 | 1.00 | 0.92 (0.60, 1.42) | 0.27 (0.10, 0.71) | 0.78 (0.50, 1.22) |
| **Colon** |  |  |  |  |
| Model 0 | 1.00 | 0.91 (0.77, 1.08) | 0.65 (0.50, 0.85) | 1.10 (0.94, 1.28) |
| Model 1 | 1.00 | 0.91 (0.77, 1.08) | 0.66 (0.50, 0.85) | 1.10 (0.94, 1.28) |
| Model 2 | 1.00 | 0.91 (0.77, 1.08) | 0.67 (0.51, 0.87) | 1.08 (0.93, 1.26) |
| Model 3 | 1.00 | 0.91 (0.77, 1.09) | 0.70 (0.52, 0.94) | 1.07 (0.91, 1.26) |
| Model 4 | 1.00 | 0.92 (0.77, 1.09) | 0.70 (0.52, 0.94) | 1.07 (0.91, 1.26) |
| Model 5 | 1.00 | 0.93 (0.78, 1.11) | 0.72 (0.53, 0.96) | 1.08 (0.91, 1.27) |
| **Rectal** |  |  |  |  |
| Model 0 | 1.00 | 0.99 (0.77, 1.27) | 0.97 (0.71, 1.34) | 0.98 (0.77, 1.25) |
| Model 1 | 1.00 | 1.00 (0.78, 1.28) | 0.99 (0.72, 1.37) | 1.01 (0.79, 1.29) |
| Model 2 | 1.00 | 0.98 (0.76, 1.25) | 1.03 (0.74, 1.42) | 0.99 (0.77, 1.26) |
| Model 3 | 1.00 | 0.96 (0.74, 1.24) | 1.09 (0.76, 1.57) | 0.92 (0.71, 1.19) |
| Model 4 | 1.00 | 0.97 (0.74, 1.25) | 1.10 (0.76, 1.58) | 0.93 (0.71, 1.20) |
| Model 5 | 1.00 | 0.97 (0.75, 1.26) | 1.11 (0.77, 1.60) | 0.93 (0.72, 1.20) |
| **Liver** |  |  |  |  |
| Model 0 | 1.00 | 0.62 (0.36, 1.08) | 0.65 (0.32, 1.35) | 1.16 (0.75, 1.77) |
| Model 1 | 1.00 | 0.63 (0.36, 1.09) | 0.67 (0.32, 1.40) | 1.14 (0.75, 1.75) |
| Model 2 | 1.00 | 0.63 (0.36, 1.10) | 0.74 (0.35, 1.55) | 1.11 (0.72, 1.70) |
| Model 3 | 1.00 | 0.57 (0.32, 1.00) | 0.70 (0.30, 1.61) | 0.92 (0.57, 1.46) |
| Model 4 | 1.00 | 0.54 (0.31, 0.96) | 0.67 (0.29, 1.56) | 0.89 (0.56, 1.43) |
| Model 5 | 1.00 | 0.55 (0.31, 0.98) | 0.70 (0.30, 1.62) | 0.90 (0.56, 1.44) |
| **Pancreas** |  |  |  |  |
| Model 0 | 1.00 | 0.97 (0.72, 1.30) | 0.77 (0.49, 1.20) | 1.09 (0.83, 1.44) |
| Model 1 | 1.00 | 1.00 (0.74, 1.34) | 0.83 (0.53, 1.29) | 1.09 (0.83, 1.44) |
| Model 2 | 1.00 | 0.98 (0.73, 1.32) | 0.85 (0.54, 1.34) | 1.08 (0.81, 1.42) |
| Model 3 | 1.00 | 0.99 (0.73, 1.35) | 0.79 (0.47, 1.31) | 1.12 (0.83, 1.51) |
| Model 4 | 1.00 | 0.98 (0.72, 1.33) | 0.78 (0.47, 1.30) | 1.11 (0.83, 1.50) |
| Model 5 | 1.00 | 1.00 (0.74, 1.36) | 0.81 (0.48, 1.34) | 1.12 (0.83, 1.51) |
| **Renal** |  |  |  |  |
| Model 0 | 1.00 | 0.61 (0.45, 0.83) | 0.55 (0.36, 0.83) | 0.94 (0.73, 1.20) |
| Model 1 | 1.00 | 0.64 (0.47, 0.87) | 0.61 (0.40, 0.92) | 0.98 (0.77, 1.26) |
| Model 2 | 1.00 | 0.63 (0.46, 0.85) | 0.63 (0.42, 0.96) | 0.97 (0.76, 1.24) |
| Model 3 | 1.00 | 0.64 (0.47, 0.88) | 0.56 (0.35, 0.89) | 0.98 (0.76, 1.28) |
| Model 4 | 1.00 | 0.65 (0.48, 0.89) | 0.57 (0.36, 0.90) | 1.00 (0.76, 1.30) |
| Model 5 | 1.00 | 0.67 (0.49, 0.92) | 0.60 (0.38, 0.96) | 1.01 (0.78, 1.32) |
| **Bladder** |  |  |  |  |
| Model 0 | 1.00 | 0.88 (0.62, 1.24) | 0.66 (0.40, 1.08) | 1.29 (0.97, 1.72) |
| Model 1 | 1.00 | 0.92 (0.65, 1.30) | 0.74 (0.45, 1.23) | 1.37 (1.03, 1.83) |
| Model 2 | 1.00 | 0.92 (0.65, 1.30) | 0.82 (0.49, 1.37) | 1.36 (1.01, 1.82) |
| Model 3 | 1.00 | 0.94 (0.66, 1.34) | 0.79 (0.44, 1.40) | 1.38 (1.01, 1.88) |
| Model 4 | 1.00 | 0.93 (0.65, 1.34) | 0.78 (0.44, 1.39) | 1.38 (1.01, 1.89) |
| Model 5 | 1.00 | 0.94 (0.66, 1.34) | 0.79 (0.44, 1.41) | 1.39 (1.01, 1.90) |
| **Thyroid** |  |  |  |  |
| Model 0 | 1.00 | 0.82 (0.55, 1.24) | 0.81 (0.43, 1.51) | 0.87 (0.57, 1.33) |
| Model 1 | 1.00 | 0.82 (0.54, 1.24) | 0.84 (0.45, 1.59) | 0.84 (0.55, 1.29) |
| Model 2 | 1.00 | 0.81 (0.53, 1.22) | 0.88 (0.47, 1.68) | 0.81 (0.53, 1.24) |
| Model 3 | 1.00 | 0.84 (0.55, 1.29) | 0.87 (0.42, 1.79) | 0.83 (0.53, 1.32) |
| Model 4 | 1.00 | 0.80 (0.52, 1.23) | 0.83 (0.40, 1.71) | 0.81 (0.51, 1.28) |
| Model 5 | 1.00 | 0.81 (0.53, 1.25) | 0.85 (0.41, 1.77) | 0.81 (0.51, 1.28) |
| **Multiple myeloma** |  |  |  |  |
| Model 0 | 1.00 | 1.23 (0.89, 1.70) | 0.53 (0.28, 0.97) | 0.91 (0.64, 1.31) |
| Model 1 | 1.00 | 1.24 (0.89, 1.71) | 0.55 (0.30, 1.02) | 0.87 (0.61, 1.25) |
| Model 2 | 1.00 | 1.23 (0.89, 1.71) | 0.55 (0.29, 1.02) | 0.87 (0.61, 1.26) |
| Model 3 | 1.00 | 1.29 (0.92, 1.81) | 0.71 (0.36, 1.39) | 0.90 (0.61, 1.33) |
| Model 4 | 1.00 | 1.28 (0.91, 1.81) | 0.71 (0.36, 1.39) | 0.90 (0.61, 1.32) |
| Model 5 | 1.00 | 1.29 (0.91, 1.82) | 0.72 (0.36, 1.41) | 0.90 (0.61, 1.32) |
| **Bronchus and lung** |  |  |  |  |
| Model 0 | 1.00 | 0.87 (0.73, 1.04) | 0.49 (0.35, 0.68) | 0.97 (0.82, 1.14) |
| Model 1 | 1.00 | 0.96 (0.81, 1.15) | 0.67 (0.48, 0.93) | 1.00 (0.85, 1.18) |
| Model 2 | 1.00 | 0.97 (0.81, 1.16) | 0.73 (0.52, 1.03) | 1.01 (0.85, 1.19) |
| Model 3 | 1.00 | 0.96 (0.79, 1.15) | 0.72 (0.49, 1.05) | 0.96 (0.80, 1.15) |
| Model 4 | 1.00 | 0.94 (0.78, 1.13) | 0.71 (0.49, 1.04) | 0.95 (0.79, 1.14) |
| Model 5 | 1.00 | 0.93 (0.78, 1.12) | 0.70 (0.48, 1.03) | 0.95 (0.79, 1.14) |
| **Malignant melanoma** |  |  |  |  |
| Model 0 | 1.00 | 0.87 (0.72, 1.05) | 1.03 (0.82, 1.30) | 0.98 (0.82, 1.18) |
| Model 1 | 1.00 | 0.86 (0.71, 1.03) | 0.97 (0.77, 1.22) | 1.01 (0.84, 1.20) |
| Model 2 | 1.00 | 0.85 (0.70, 1.03) | 0.92 (0.73, 1.16) | 1.01 (0.84, 1.21) |
| Model 3 | 1.00 | 0.87 (0.72, 1.06) | 0.96 (0.74, 1.24) | 1.05 (0.87, 1.27) |
| Model 4 | 1.00 | 0.87 (0.72, 1.06) | 0.96 (0.74, 1.25) | 1.05 (0.87, 1.27) |
| Model 5 | 1.00 | 0.88 (0.72, 1.07) | 0.97 (0.74, 1.25) | 1.06 (0.87, 1.27) |

^a^walking only or combined it with either car or public transport or both, ^b^cycling only or combined it with any other mode/s

^c^public transport alone or combined it with car

Model 0: stratified by age group, gender, region, Townsend deprivation index

Model 1: Model 0 + Ethnicity + education + smoking + alcohol + vegetables + processed meat

Model 2: Model 1 + stairs climbed frequency + walk for pleasure + other exercise + strenuous sports + light DIY + heavy DIY + Walk/Stand in job + heavy manual/physical work in job + sedentary time

Model 3: Model 2 + transport modes for non-commute trips

Model 4: Model 3 + environmental factors (PM_2.5_, NO_2_, greenspace)

Model 5: Model 4 + BMI

Table S5: Results for female-specific cancers

|  | **Hazard ratio (95% CI)** | | | |
| --- | --- | --- | --- | --- |
|  | **Car only** | **Walk**^a^ | **Cycle**^b^ | **Public Transport**^c^ |
| **Breast** |  |  |  |  |
| Model 0 | 1.00 | 0.95 (0.87, 1.03) | 0.88 (0.76, 1.02) | 1.01 (0.93, 1.10) |
| Model 1 | 1.00 | 0.95 (0.88, 1.04) | 0.86 (0.74, 0.99) | 1.03 (0.94, 1.12) |
| Model 2 | 1.00 | 0.95 (0.88, 1.04) | 0.87 (0.75, 1.02) | 1.01 (0.93, 1.11) |
| Model 3 | 1.00 | 0.96 (0.88, 1.05) | 0.86 (0.72, 1.02) | 1.03 (0.94, 1.13) |
| Model 4 | 1.00 | 0.95 (0.87, 1.04) | 0.85 (0.72, 1.01) | 1.02 (0.93, 1.12) |
| Model 5 | 1.00 | 0.96 (0.88, 1.04) | 0.86 (0.72, 1.02) | 1.02 (0.93, 1.12) |
| **Breast (postmenopausal)** |  |  |  |  |
| Model 0 | 1.00 | 0.93 (0.83, 1.05) | 0.78 (0.62, 1.00) | 1.00 (0.89, 1.13) |
| Model 1 | 1.00 | 0.94 (0.83, 1.06) | 0.79 (0.62, 1.00) | 1.02 (0.90, 1.15) |
| Model 2 | 1.00 | 0.95 (0.84, 1.07) | 0.81 (0.63, 1.03) | 1.00 (0.89, 1.14) |
| Model 3 | 1.00 | 0.95 (0.84, 1.07) | 0.85 (0.64, 1.12) | 1.01 (0.89, 1.15) |
| Model 4 | 1.00 | 0.94 (0.83, 1.07) | 0.84 (0.64, 1.11) | 1.01 (0.89, 1.15) |
| Model 5 | 1.00 | 0.95 (0.84, 1.08) | 0.85 (0.64, 1.12) | 1.01 (0.89, 1.15) |
| **Endometrial** |  |  |  |  |
| Model 0 | 1.00 | 0.82 (0.65, 1.03) | 0.68 (0.44, 1.06) | 0.93 (0.74, 1.17) |
| Model 1 | 1.00 | 0.81 (0.64, 1.02) | 0.68 (0.43, 1.06) | 0.93 (0.74, 1.17) |
| Model 2 | 1.00 | 0.83 (0.66, 1.04) | 0.73 (0.46, 1.15) | 0.91 (0.72, 1.15) |
| Model 3 | 1.00 | 0.88 (0.69, 1.12) | 0.65 (0.39, 1.08) | 0.98 (0.76, 1.25) |
| Model 4 | 1.00 | 0.89 (0.70, 1.14) | 0.66 (0.39, 1.10) | 0.99 (0.77, 1.27) |
| Model 5 | 1.00 | 0.94 (0.74, 1.20) | 0.72 (0.43, 1.20) | 1.01 (0.79, 1.29) |
| **Ovarian** |  |  |  |  |
| Model 0 | 1.00 | 0.99 (0.75, 1.30) | 0.80 (0.47, 1.37) | 0.98 (0.74, 1.31) |
| Model 1 | 1.00 | 0.98 (0.74, 1.30) | 0.79 (0.46, 1.35) | 1.00 (0.75, 1.33) |
| Model 2 | 1.00 | 0.99 (0.75, 1.31) | 0.79 (0.46, 1.35) | 1.00 (0.75, 1.34) |
| Model 3 | 1.00 | 0.99 (0.74, 1.32) | 0.64 (0.35, 1.18) | 0.99 (0.73, 1.35) |
| Model 4 | 1.00 | 0.99 (0.74, 1.33) | 0.64 (0.35, 1.18) | 0.99 (0.73, 1.35) |
| Model 5 | 1.00 | 1.00 (0.74, 1.34) | 0.65 (0.35, 1.20) | 1.00 (0.73, 1.36) |

^a^walking only or combined it with either car or public transport or both, ^b^cycling only or combined it with any other mode/s

^c^public transport alone or combined it with car;

adjustments in the models 0-5 are the same as those adjusted in the models 0-5 in Table 4

Table S6: Results for female-specific cancers by additionally adjusting for reproductive health factors

|  | **Hazard ratio (95% CI)** | | | |
| --- | --- | --- | --- | --- |
|  | **Car only** | **Walk**^a^ | **Cycle**^b^ | **Public Transport**^c^ |
| **Breast** | 1.00 | 0.96 (0.87, 1.04) | 0.85 (0.72, 1.01) | 1.02 (0.93, 1.12) |
| **Endometrial** | 1.00 | 0.92 (0.72, 1.18) | 0.69 (0.41, 1.16) | 0.97 (0.75, 1.24) |
| **Ovarian** | 1.00 | 1.00 (0.75, 1.34) | 0.65 (0.35, 1.20) | 0.97 (0.71, 1.32) |

^a^walking only or combined it with either car or public transport or both, ^b^cycling only or combined it with any other mode/s

^c^public transport alone or combined it with car; reproductive health factors adjusted: menopausal status, parity, use of oral contraceptive pills, use of hormone replacement therapy

Table S7: Results for male-specific cancer (prostate cancer)

|  | **Hazard ratio (95% CI)** | | | |
| --- | --- | --- | --- | --- |
| **Prostate** | **Car only** | **Walk**^a^ | **Cycle**^b^ | **Public transport**^c^ |
| Model 0 | 1.00 | 1.00 (0.90, 1.11) | 0.98 (0.87, 1.10) | 1.13 (1.03, 1.24) |
| Model 1 | 1.00 | 0.99 (0.89, 1.10) | 0.97 (0.86, 1.08) | 1.09 (0.99, 1.19) |
| Model 2 | 1.00 | 0.99 (0.89, 1.09) | 0.96 (0.85, 1.08) | 1.08 (0.98, 1.18) |
| Model 3 | 1.00 | 1.00 (0.90, 1.11) | 0.93 (0.82, 1.07) | 1.09 (0.99, 1.21) |
| Model 4 | 1.00 | 1.00 (0.90, 1.11) | 0.94 (0.82, 1.07) | 1.09 (0.99, 1.21) |
| Model 5 | 1.00 | 0.99 (0.89, 1.11) | 0.93 (0.81, 1.06) | 1.09 (0.99, 1.20) |

^a^walking only or combined it with either car or public transport or both, ^b^cycling only or combined it with any other mode/s

^c^public transport alone or combined it with car

adjustments in the models 0-5 are the same as those adjusted in the models 0-5 in Table 4

## Sub-group analyses

**By gender**

Table S8: Results for subgroup analyses by gender for cancers with >10 cases per exposure group

|  | **Hazard ratio (95% CI)**^a^ | | | |  |
| --- | --- | --- | --- | --- | --- |
| **Cancer** | **Car only** | **Walk**^b^ | **Cycle**^c^ | **Public transport^d^** | **p value**^e^ |
| **Colon** |  |  |  |  |  |
| Women | 1.00 | 0.93 (0.73, 1.17) | 0.76 (0.45, 1.27) | 1.11 (0.88, 1.40) | 0.86 |
| Men | 1.00 | 0.93 (0.72, 1.22) | 0.69 (0.48, 0.99) | 1.05 (0.83, 1.32) |  |
| **Rectal** |  |  |  |  |  |
| Women | 1.00 | 1.22 (0.83, 1.81) | 1.72 (0.90, 3.27) | 1.18 (0.78, 1.78) | 0.13 |
| Men | 1.00 | 0.84 (0.59, 1.20) | 0.92 (0.58, 1.44) | 0.81 (0.58, 1.14) |  |
| **Bronchus and lung** |  |  |  |  |  |
| Women | 1.00 | 1.03 (0.81, 1.31) | 1.07 (0.60, 1.88) | 0.94 (0.73, 1.22) | 0.23 |
| Men | 1.00 | 0.84 (0.62, 1.14) | 0.54 (0.32, 0.90) | 0.99 (0.75, 1.29) |  |
| **Malignant melanoma** |  |  |  |  |  |
| Women | 1.00 | 0.89 (0.70, 1.14) | 0.78 (0.50, 1.23) | 0.99 (0.76, 1.30) | 0.54 |
| Men | 1.00 | 0.79 (0.57, 1.10) | 1.09 (0.79, 1.50) | 1.11 (0.85, 1.44) |  |

^a^result of the fully adjusted model

^b^walking only or combined it with either car or public transport or both, ^c^cycling only or combined it with any other mode/s

^d^public transport alone or combined it with car

^e^p value for likelihood ratio test for the models with and without interaction terms for commuting modes by gender (degrees of freedom = 3)

**By smoking status**

Table S9: Results for subgroup analyses by smoking status for cancers with >10 cases per exposure group

|  | **Hazard ratio (95% CI)**^a^ | | | |  |
| --- | --- | --- | --- | --- | --- |
| **Cancer** | **Car only** | **Walk**^b^ | **Cycle**^c^ | **Public transport^d^** | **p value**^e^ |
| **Colon** |  |  |  |  |  |
| Never | 1.00 | 1.17 (0.89, 1.53) | 0.59 (0.36, 0.98) | 1.15 (0.88, 1.49) | 0.52 |
| Previous and current | 1.00 | 0.82 (0.65, 1.03) | 0.80 (0.56, 1.15) | 1.04 (0.84, 1.29) |  |
| **Rectal** |  |  |  |  |  |
| Never | 1.00 | 0.82 (0.53, 1.28) | 1.63 (0.95, 2.80) | 0.73 (0.46, 1.15) | 0.20 |
| Previous and current | 1.00 | 1.06 (0.77, 1.47) | 0.85 (0.52, 1.39) | 1.06 (0.77, 1.45) |  |
| **Malignant melanoma** |  |  |  |  |  |
| Never | 1.00 | 0.83 (0.62, 1.12) | 0.81 (0.52, 1.25) | 1.07 (0.80, 1.44) | 0.56 |
| Previous and current | 1.00 | 0.91 (0.70, 1.17) | 1.07 (0.77, 1.48) | 1.05 (0.82, 1.35) |  |
| **Gender specific** |  |  |  |  |  |
| **Breast** |  |  |  |  |  |
| Never | 1.00 | 0.92 (0.81, 1.05) | 0.78 (0.59, 1.03) | 0.98 (0.85, 1.13) | 0.69 |
| Previous and current | 1.00 | 0.98 (0.87, 1.11) | 0.91 (0.73, 1.13) | 1.05 (0.93, 1.19) |  |
| **Endometrial** |  |  |  |  |  |
| Never | 1.00 | 0.82 (0.58, 1.15) | 0.73 (0.35, 1.54) | 0.89 (0.63, 1.26) | 0.75 |
| Previous and current | 1.00 | 1.11 (0.78, 1.57) | 0.71 (0.34, 1.46) | 1.14 (0.79, 1.63) |  |
| **Prostate** |  |  |  |  |  |
| Never | 1.00 | 0.83 (0.68, 1.00) | 0.92 (0.74, 1.14) | 0.98 (0.83, 1.15) | 0.11 |
| Previous and current | 1.00 | 1.08 (0.94, 1.23) | 0.93 (0.78, 1.10) | 1.16 (1.03, 1.31) |  |

^a^result of the fully adjusted model

^b^walking only or combined it with either car or public transport or both, ^c^cycling only or combined it with any other mode/s

^d^public transport alone or combined it with car

^e^p value for likelihood ratio test for the models with and without interaction terms for commuting modes by smoking status (degrees of freedom = 3)

**By environmental factors**

Breast, colon, bronchus, and lung cancers, which were previously shown to have an association with air pollution and had a relatively large number of cases in this study, were included in the analyses.

**PM_2.5_**

Table S10: Results for subgroup analyses by PM_2.5_ levels

|  | **Hazard ratio (95% CI)**^a^ | | | |  |
| --- | --- | --- | --- | --- | --- |
| **Cancer** | **Car only** | **Walk**^b^ | **Cycle**^c^ | **Public transport^d^** | **p value**^e^ |
| **Colon** |  |  |  |  |  |
| Lower than threshold | 1.00 | 1.00 (0.76, 1.31) | 0.82 (0.54, 1.26) | 1.16 (0.90, 1.49) | 0.56 |
| Higher than threshold | 1.00 | 0.84 (0.65, 1.08) | 0.59 (0.37, 0.94) | 0.91 (0.70, 1.17) |  |
| **Bronchus and lung** |  |  |  |  |  |
| Lower than threshold | 1.00 | 1.01 (0.73, 1.39) | 0.57 (0.29, 1.11) | 0.91 (0.65, 1.27) | 0.69 |
| Higher than threshold | 1.00 | 0.92 (0.72, 1.18) | 0.80 (0.49, 1.32) | 1.00 (0.78, 1.30) |  |
| **Breast (women)** |  |  |  |  |  |
| Lower than threshold | 1.00 | 0.97 (0.84, 1.11) | 0.81 (0.62, 1.06) | 1.00 (0.87, 1.16) | 0.68 |
| Higher than threshold | 1.00 | 0.91 (0.80, 1.03) | 0.86 (0.67, 1.10) | 1.06 (0.92, 1.21) |  |

^a^result of the fully adjusted model

^b^walking only or combined it with either car or public transport or both, ^c^cycling only or combined it with any other mode/s

^d^public transport alone or combined it with car

^e^p value for likelihood ratio test for the models with and without interaction terms for commuting modes by PM_2.5_ level (degrees of freedom = 3)

**Nitrogen dioxide (NO_2_)**

Table S11: Results for subgroup analyses by NO_2_ levels

|  | **Hazard ratio (95% CI)**^a^ | | | |  |
| --- | --- | --- | --- | --- | --- |
| **Cancer** | **Car only** | **Walk**^b^ | **Cycle**^c^ | **Public transport^d^** | **p value**^e^ |
| **Colon** |  |  |  |  |  |
| Lower than threshold | 1.00 | 0.86 (0.63, 1.18) | 0.81 (0.51, 1.28) | 1.25 (0.96, 1.62) | 0.44 |
| Higher than threshold | 1.00 | 0.95 (0.77, 1.19) | 0.67 (0.45, 0.99) | 0.99 (0.80, 1.23) |  |
| **Bronchus and lung** |  |  |  |  |  |
| Lower than threshold | 1.00 | 0.85 (0.60, 1.21) | 0.52 (0.25, 1.07) | 0.72 (0.49, 1.06) | 0.45 |
| Higher than threshold | 1.00 | 0.96 (0.77, 1.20) | 0.76 (0.48, 1.21) | 1.00 (0.80, 1.25) |  |
| **Breast (women)** |  |  |  |  |  |
| Lower than threshold | 1.00 | 1.23 (1.06, 1.42) | 0.69 (0.53, 0.90) | 0.94 (0.80, 1.12) | 0.07 |
| Higher than threshold | 1.00 | 1.00 (0.89, 1.12) | 0.69 (0.55, 0.85) | 1.13 (1.01, 1.27) |  |

^a^result of the fully adjusted model

^b^walking only or combined it with either car or public transport or both, ^c^cycling only or combined it with any other mode/s

^d^public transport alone or combined it with car

^e^p value for likelihood ratio test for the models with and without interaction terms for commuting modes by NO_2_ level (degrees of freedom = 3)

**Greenspace percentage**

Table S12: Results for subgroup analyses by residential greenspace levels

|  | **Hazard ratio (95% CI)**^a^ | | | |  |
| --- | --- | --- | --- | --- | --- |
| **Cancer** | **Car only** | **Walk**^b^ | **Cycle**^c^ | **Public transport^d^** | **p value**^e^ |
| **Colon** |  |  |  |  |  |
| Lower than threshold | 1.00 | 1.11 (0.87, 1.41) | 0.79 (0.52, 1.20) | 1.07 (0.84, 1.38) | 0.17 |
| Higher than threshold | 1.00 | 0.67 (0.49, 0.93) | 0.57 (0.36, 0.91) | 1.07 (0.83, 1.38) |  |
| **Bronchus and lung** |  |  |  |  |  |
| Lower than threshold | 1.00 | 0.89 (0.69, 1.15) | 0.66 (0.39, 1.11) | 0.95 (0.73, 1.23) | 0.93 |
| Higher than threshold | 1.00 | 1.03 (0.76, 1.40) | 0.44 (0.24, 0.83) | 0.98 (0.73, 1.34) |  |
| **Breast (women)** |  |  |  |  |  |
| Lower than threshold | 1.00 | 0.85 (0.74, 0.96) | 0.87 (0.68, 1.10) | 0.99 (0.87, 1.14) | 0.44 |
| Higher than threshold | 1.00 | 1.02 (0.89, 1.17) | 0.84 (0.65, 1.09) | 1.02 (0.88, 1.18) |  |

^a^result of the fully adjusted model

^b^walking only or combined it with either car or public transport or both, ^c^cycling only or combined it with any other mode/s

^d^public transport alone or combined it with car

^e^p value for likelihood ratio test for the models with and without interaction terms for commuting modes by greenspace level (degrees of freedom = 3)

**Walking, Cycling and Public transport-only modes**

Table S13: Results for cancers in both genders (individual specific modes)

|  | **Hazard ratio (95% CI)^a^** | | | |
| --- | --- | --- | --- | --- |
|  | **Car only (ref)** | **Walk only** | **Cycle only** | **Public transport only** |
| **Both genders** |  |  |  |  |
| Colon | 1.00 | 1.14 (0.89, 1.45) | 0.94 (0.59, 1.49) | 1.10 (0.89, 1.37) |
| Bronchus and lung | 1.00 | 0.99 (0.77, 1.27) | 0.58 (0.29, 1.15) | 1.07 (0.86, 1.33) |
| Malignant melanoma | 1.00 | 0.95 (0.71, 1.27) | 1.15 (0.75, 1.74) | 1.00 (0.77, 1.30) |
| **Gender-specific cancers** |  |  |  |  |
| Breast (women) | 1.00 | 0.91 (0.80, 1.04) | 0.82 (0.60, 1.13) | 1.00 (0.89, 1.13) |
| Prostate (men) | 1.00 | 1.09 (0.92, 1.29) | 0.81 (0.65, 1.02) | 1.14 (1.00, 1.30) |

^a^result of the fully adjusted model

**Reverse causation**

Table S14: Results for analyses by removing the first four years of follow-up

|  | **Hazard ratio (95% CI)^a^** | | | |
| --- | --- | --- | --- | --- |
|  | **Car only (ref)** | **Walk**^b^ | **Cycle**^c^ | **Public transport^d^** |
| **Both genders** |  |  |  |  |
| Oesophagus (adenocarcinoma) | 1.00 | 1.09 (0.65, 1.83) | 0.48 (0.18, 1.31) | 1.20 (0.75, 1.94) |
| Stomach | 1.00 | 1.03 (0.63, 1.67) | **0.34 (0.11, 0.99)** | 0.76 (0.45, 1.29) |
| Colon | 1.00 | 0.87 (0.70, 1.08) | **0.70 (0.49, 0.99)** | 1.09 (0.89, 1.32) |
| Rectal | 1.00 | 0.96 (0.70, 1.31) | 1.24 (0.82, 1.90) | 1.02 (0.75, 1.37) |
| Liver | 1.00 | 0.88 (0.61, 1.26) | 0.71 (0.39, 1.29) | 1.12 (0.80, 1.57) |
| Pancreas | 1.00 | 0.88 (0.61, 1.26) | 0.71 (0.39, 1.29) | 1.12 (0.80, 1.57) |
| Renal | 1.00 | **0.66 (0.45, 0.95)** | 0.65 (0.39, 1.08) | 1.05 (0.77, 1.43) |
| Bladder | 1.00 | 1.05 (0.69, 1.59) | 0.87 (0.44, 1.69) | 1.29 (0.88, 1.91) |
| Thyroid | 1.00 | 0.99 (0.61, 1.59) | 0.86 (0.36, 2.03) | 0.83 (0.48, 1.43) |
| Multiple myeloma | 1.00 | 1.32 (0.89, 1.95) | 0.80 (0.38, 1.70) | 0.87 (0.56, 1.36) |
| Bronchus and lung | 1.00 | 0.94 (0.76, 1.16) | 0.80 (0.52, 1.23) | 0.81 (0.65, 1.02) |
| Malignant melanoma | 1.00 | 0.84 (0.67, 1.06) | 0.94 (0.69, 1.28) | 1.02 (0.81, 1.27) |
| **Gender specific** |  |  |  |  |
| **Women** |  |  |  |  |
| Breast | 1.00 | 0.98 (0.88, 1.10) | 0.87 (0.71, 1.08) | 0.99 (0.88, 1.11) |
| Endometrial | 1.00 | 0.85 (0.63, 1.14) | 0.82 (0.45, 1.51) | 0.97 (0.72, 1.31) |
| Ovarian | 1.00 | 1.09 (0.77, 1.56) | 0.81 (0.37, 1.77) | 1.17 (0.81, 1.70) |
| **Men** |  |  |  |  |
| Prostate | 1.00 | 1.01 (0.89, 1.14) | 0.97 (0.84, 1.13) | 1.08 (0.96, 1.21) |

^a^result of the fully adjusted model

^b^walking only or combined it with either car or public transport or both, ^c^cycling only or combined it with any other mode/s

^d^public transport alone or combined it with car

Table S15: Sensitivity analyses by adjusting long-standing illness additionally

|  | **Hazard ratio (95% CI)^a^** | | | |
| --- | --- | --- | --- | --- |
|  | **Car only (ref)** | **Walk**^b^ | **Cycle**^c^ | **Public transport^d^** |
| **Both genders** |  |  |  |  |
| Oesophagus (adenocarcinoma) | 1.00 | 1.07 (0.68, 1.68) | 0.64 (0.29, 1.41) | 1.36 (0.92, 2.03) |
| Stomach | 1.00 | 0.92 (0.60, 1.42) | **0.27 (0.10, 0.71)** | 0.78 (0.50, 1.22) |
| Colon | 1.00 | 0.93 (0.78, 1.11) | **0.72 (0.53, 0.96)** | 1.08 (0.91, 1.27) |
| Rectal | 1.00 | 0.97 (0.75, 1.26) | 1.11 (0.77, 1.60) | 0.93 (0.72, 1.20) |
| Liver | 1.00 | **0.55 (0.31, 0.98)** | 0.70 (0.30, 1.63) | 0.88 (0.55, 1.41) |
| Pancreas | 1.00 | 1.00 (0.73, 1.35) | 0.81 (0.48, 1.34) | 1.12 (0.83, 1.51) |
| Renal | 1.00 | **0.67 (0.49, 0.92)** | **0.60 (0.38, 0.96)** | 1.01 (0.77, 1.32) |
| Bladder | 1.00 | 0.94 (0.66, 1.35) | 0.79 (0.44, 1.41) | 1.39 (1.01, 1.90) |
| Thyroid | 1.00 | 0.81 (0.53, 1.25) | 0.85 (0.41, 1.77) | 0.80 (0.51, 1.27) |
| Multiple myeloma | 1.00 | 1.29 (0.92, 1.82) | 0.72 (0.36, 1.41) | 0.90 (0.61, 1.33) |
| Bronchus and lung | 1.00 | 0.93 (0.78, 1.12) | 0.70 (0.48, 1.03) | 0.94 (0.78, 1.13) |
| Malignant melanoma | 1.00 | 0.88 (0.72, 1.07) | 0.96 (0.74, 1.25) | 1.06 (0.88, 1.28) |
| **Gender specific** |  |  |  |  |
| **Women** |  |  |  |  |
| Breast | 1.00 | 0.95 (0.87, 1.04) | 0.85 (0.72, 1.01) | 1.02 (0.93, 1.12) |
| Endometrial | 1.00 | 0.94 (0.74, 1.20) | 0.71 (0.42, 1.20) | 1.01 (0.79, 1.29) |
| Ovarian | 1.00 | 1.00 (0.74, 1.34) | 0.65 (0.35, 1.20) | 1.00 (0.73, 1.36) |
| **Men** |  |  |  |  |
| Prostate | 1.00 | 0.99 (0.89, 1.11) | 0.93 (0.81, 1.06) | 1.09 (0.99, 1.20) |

^a^adjusted the covariates in the model 5 and long-standing illness

^b^walking only or combined it with either car or public transport or both, ^c^cycling only or combined it with any other mode/s

^d^public transport alone or combined it with car
